# Short-term survival and mortality rates in a retrospective study of colic in 1588 Danish horses

**DOI:** 10.1186/1751-0147-56-20

**Published:** 2014-04-08

**Authors:** Mogens T Christophersen, Nana Dupont, Kristina S Berg-Sørensen, Christel Konnerup, Tina H Pihl, Pia H Andersen

**Affiliations:** 1 Department of Large Animal Sciences, Faculty of Health and Medical Sciences, University of Copenhagen, Hoejbakkegaard Alle 5, Taastrup DK-2630, Denmark; 2 Department of Clinical Sciences, Swedish Agricultural University, Box 7054, Uppsala SE-75007, Sweden

**Keywords:** Equine colic, Surgery, Survival, Euthanasia, Disease severity score, Quality of care

## Abstract

**Background:**

Outcomes of colic treatment are of great interest to clinicians, horse owners and insurers. One commonly used criterion of success is the overall short-term survival rate. This is used as to compare treatments and to measure quality of veterinary care, but may be biased by demographic or social factors such as attitudes towards animal suffering and euthanasia. The aims of this study were to 1) describe and analyse characteristics in horses with signs of colic referred to the University Hospital for Large Animals (UHLA), University of Copenhagen, Denmark over a 10-year period and 2) to compare these rates with those published in other comparable studies.

**Results:**

The overall survival rate for colic horses over the 10-year study period was 68% (confidence intervals (CI): 66–71%; 1087/1588). In the medical group, 1093 horses, short-term survival was 87% (CI: 85–89%). Thirty one % of referred horses were given diagnoses requiring surgical intervention (CI: 29–33%). In this group 32% of the horses were euthanized before surgery (CI: 28–36%; 159/495). Of the surgical cases 27% (CI: 23-31%) were euthanized or died during surgery. Of the horses that recovered from surgery 25% died or were euthanized (CI: 19–32%; 48/189), while 75% survived to discharge (CI: 68–81%).

**Conclusions:**

The short term survival rates of Danish horses with colic were similar or lower to those reported from other countries. Apart from variability of veterinary care, attitudes towards euthanasia vary among the countries, which may bias the outcomes. This study indicates that qualitative interview studies on owners’ attitudes towards animal suffering and euthanasia need to be conducted. Our opinion is that survival rates are not valid as sole indicators of quality of care in colic treatment due to selection bias. If the survival rates are to be compared between hospitals, techniques or surgeons, prospective studies including mutually agreed-on disease severity scores and a predefined set of reasons for euthanasia are needed.

## Introduction

**Figure 1:**
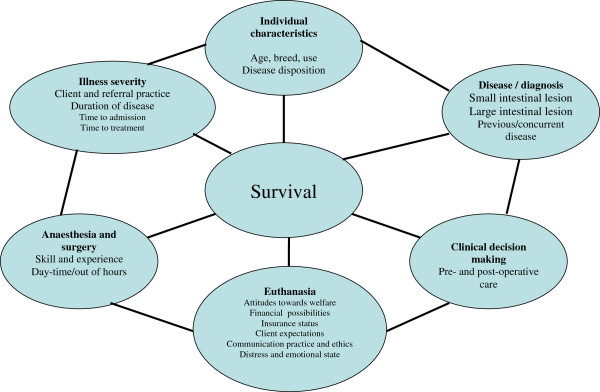
Some factors determining colic survival, and influencing comparison between different equine hospitals.

Despite an improving trend, the high level of mortality and frequent complications of surgery for equine colic is of concern to equine surgeons and those owning or working with horses and the insurers, given that close to 10% of horses with colic require surgery [1]. Equine clinicians need information on mortality rates from countries or regions with similar criteria for choosing treatment or euthanasia as the clinician uses in order to provide accurate advice to clients on prognosis and costs and to continue to develop and improve his or her standards of colic treatment. However, the differing criteria for selection of study populations in the mainly retrospective studies [2] can make comparisons of treatment outcomes difficult to compare. One commonly used criterion of success is the overall short-term survival rate, defined as the rate of horses with diagnosed colic discharged from the clinic. Colic is a complex, multifactorial disease. Its short-term survival therefore may depend on a number of factors impacting the horse population at risk (Figure 1). Apart from the veterinary care, survival rate is also affected by demographic (Reeves *et al*. 1989) [3] or social factors, such as attitudes towards animal suffering and euthanasia. In view of this, survival rates may not always provide meaningful information on the quality of care – and this is especially so where euthanasia is an accepted option. If treatment of colic is to improve, criteria allowing comparisons among hospitals and practices need to be made [4]. This paper categorises and analyses factors affecting overall, medical and surgical short-term survival rates for equine colic cases and examines a number of population characteristics in horses with signs of colic referred to the University Hospital for Large Animals (UHLA), University of Copenhagen, Denmark, over a 10-year period. We also aimed to analyse the frequency of, and reasons for, euthanasia in the UHLA surgical cases. Finally, we compared these rates with those obtained in comparable studies from other countries (Table 1).

**Table 1:** Distribution of cases, mortality rates and short-term survival rates from several international studies

**Author**	**Year, Country**	**Investigation period**	**No. of colic cases**	**Distribution of cases**		**Cases euthanized or dead %**				**Short-term survival rates (%)**		
				**Medical %**	**Surgical %**	**Without any treatment**	**Medical cases**	**Cases undergoing surgery**				
								**During surgery**	**After recovery**	**Overall**	**Medically treated cases**	**Surgically treated cases**
[3]	1989, USA	1974-1984	320	41	59	16	6	31	25	59	94	44
[7]	2005, Germany	1990-1997	1431	80	20	*	7	35	16	84	93	49
[5]	2003, The Netherlands	1999-2000	649	64	36	8	15	26	21	69	85	54
[6]	2005, Canada	1992-2002	604	54	46	21	6	26	14	66	94	60
[8]	2009, Israel	2003-2006	208	30	70	6	8**	34		71	92	66
[9]	2009, South Africa	1998-2007	929	60	40	3	7	21	13	79	93	66
UHLA***	2013, Denmark	2000-2009	1588	69	31	10	13	44	14	68	87	42

*Data not given.

**Cases not following the recommendation of surgery were excluded.

***This is data of the present study.

## Materials and methods

### Case selection

Prior to hospitalization at UHLA, the horses had been examined by a referring veterinarian on one or several occasions. Medical records from 1st January 2000 and 31st December 2009 of all horses suspected to have colic aged >1 year were reviewed and variables relating to the history and signalment of the horse (age, breed, sex, initial diagnosis on referral, referral date); for diagnosis and treatment of the colic (date of surgery, clinical diagnosis, surgical diagnosis including disease location); and for outcome (date of discharge, death or euthanasia, and pathological diagnosis) were extracted. To improve consistency of assignment of data the consensus decisions of three authors (MTC, THP and PHA) reviewing the hospital records together were used. When they disagreed, the decision of the first author of the study was used. Inclusion criteria were: gastric reflux and signs of abdominal discomfort or pain (including pawing, sweating, tooth-grinding, stretching, lip curling, looking and/or kicking at the abdomen, rolling and lying down); with or without peritoneal fluid abnormality or rectal palpation abnormalities. Horses either dead or moribund on arrival to the hospital were excluded, as were horses with a primary diagnosis of oesophageal obstruction, diarrhoea without signs of colic, gastric ulcers without signs of colic, or inguinal, diaphragmatic or umbilical hernia. For horses with more than one hospitalization due to colic and horses with re-laparotomy due to colic, only the first time was included. Decisions on surgery were made by the surgeon on call. Generally, however, they were based on two or more of the following criteria: persistent gastric reflux of more than 2 L, refractory to pain management, absence of borborygmi, findings on rectal palpation consistent with volvulus, cardiovascular and systemic abnormalities, elevated plasma lactate concentration, peritoneal fluid with protein concentrations > 25 g per L and/or abnormal contents such as white blood cells or haemolysis. Horses not fulfilling the criteria for surgery were treated medically. If a decision was changed from medical to surgical treatment during hospitalization, only the final assignment was recorded. To describe the hospital population as a whole, data on the age, breed (thoroughbred, standardbred, warmblood, light draft, draft, Icelandic horses, ponies and mixed breeds) and gender of all horses admitted to the UHLA during the period 2005–2009 were obtained from the medical record. The client communication records were screened for qualitative information on the decisions made. Statements on owner’s preferences were noted before, during and after colic surgery.

We compared our results with a number of other studies selected by applying the following criteria: all colic horses admitted to a university hospital during a certain period were included; a large proportion of horses were referred cases; medical, surgical, dead and euthanized cases could be identified; and the specific short-term survival and mortality rates for medical and surgical colic cases could be calculated. For ease of comparison, the data of the present study were included as "UHLA".

### Statistical methods

The number of horses surviving to the point of discharge in the medical and surgical group was used to calculate overall short-term survival rates. In the surgical cases this rate was calculated as the number of horses that underwent surgery divided by the number of horses discharged from the hospital. Mortality for horses in the surgical group was noted at several critical control points: pre-operatively, intra-operatively and post-operatively. Descriptive parameters were evaluated non-statistically. Survival rate percentages, standard error (SE) and 95% confidence intervals (CIs) were calculated using R 3.0.0 for Windows. A test for normality of age distribution was conducted using the D’Agostino and Pearson omnibus test. Age distribution was assessed using the Z test. The variables gender and breed distribution were tested using Chi-square (χ^2^) or Fishers exact test, as were comparisons with other studies. All levels of significance were set to p < 0.05.

## Results

**Figure 2:**
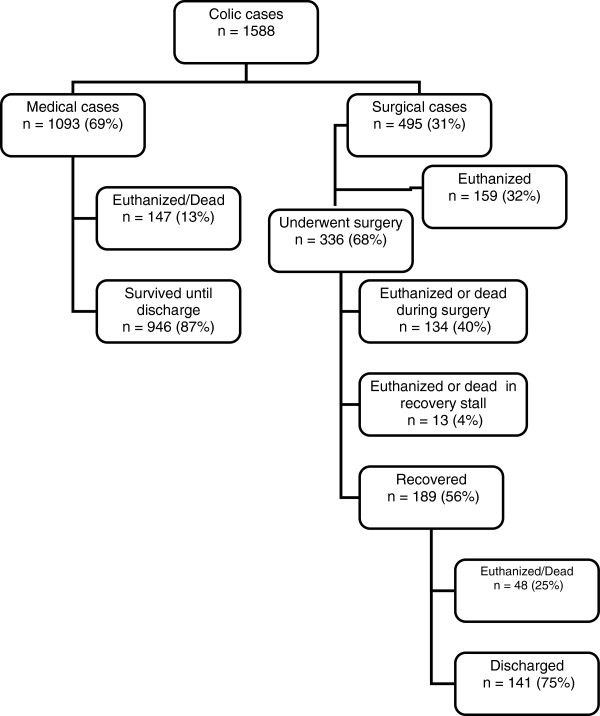
Outcome of colic treatments of 1588 colic cases referred to the University Hospital for Large Animals, Denmark in 2000–2009.

The records of 1588 horses remained for analysis. Mean age of total study population was 9.4 years (range 1–36). The number of horses allocated to medical and surgical treatment and the subsequent outcomes are shown in Figure 2. The overall survival rate for colic horses over the 10-year study period was 68% (SE: 1.2%; CI: 66–71%; 1087/1588). In the medical group, 1093 horses, short-term survival was 87% (SE: 1.0%; CI: 85–89%; 946/1093). In all 31% of referred horses were given diagnoses requiring surgical intervention (SE: 1.2%; CI: 29–33%; 495/1588). In this group 32% of the horses were euthanized before surgery (SE: 2.1%; CI: 28–36%; 159/495). Of the surgical cases 27% were euthanized or died during surgery (SE: 2.0%; CI: 23-31%; 134/495). Three per cent were euthanized or died in the padded recovery box after surgery (SE 0.7%; CI: 1-4%; 13/495). Of the horses that recovered from surgery 25% died or were euthanized (SE: 3.2%; CI: 19–32%; 48/189), while 75% survived to discharge (SE: 3.2%; CI: 68–81%; 141/189). Surgical short-term survival rates varied from 28% of all horses with a surgical diagnosis, to 42% of horses that underwent surgery, to 75% of horses that were allowed to recover (Figure 2).

Short-term survival was grouped according to diagnosis (Table 2). No statistical significant difference in survival rates among mares, geldings and stallions were observed (data are not shown). Tympanic colic and simple obstruction and displacement of colon had high survival rates, 94%, 88% and 89% respectively, while ruptured viscus had a low survival rate (2.7%). Short-term survival was grouped according to localization and type of lesion (Table 3). 54% (SE: 1.3%; CI: 51–56%; 852/1588) of the colic horses had a lesion localized to the large intestine and 14% (SE: 0.9%; CI: 13–16%; 230/1588) had a small intestinal lesion. The reason for gastrointestinal pain remained unexplained during hospitalization in 19% of all colic horses (SE: 0.9%; CI: 17–21%). During the screening of the client communication records it became obvious that it was impossible to discriminate between the owner’s financial constraints, the owner’s perception of the welfare of the horse, the owner’s plans to make future use of the horse, and the surgeon’s advice on the decision. Reasons for euthanasia before surgery included financial constraints, in some cases linked to lack of insurance, concerns regarding suffering of the horse, high age, poor prognosis of recovery and anticipation of weak athletic performance postoperatively. Reasons for euthanasia during surgery included financial constraints, often in combination with the relevant prognosis. Reasons for euthanasia after recovery, but before discharge, included post-operative complications such as shock, severe pain/recurrent colic, laminitis and, on a single occasion, myositis.

**Table 2:** Diagnosis, treatment category and short-term outcome in 1588 colic horses referred to the University Hospital for Large Animals, Denmark, 2000–2009

		**Survival to discharge**		**Euthanized or dead before discharge**	
Diagnosis	N	Medical	Surgical	Medical	Surgical
Large colon/caecum impaction	360	301	15	11	33
Undiagnosed	302	269	4	5	24
Large colon displacement	274	190	54	3	27
Small intestine: Volvulus/intussusception/strangulation	162	3	35	7	117
Tympany	87	80	2	0	5
Large colon: Volvulus/intussusception/strangulation	71	1	9	9	52
Ruptured viscus	74	2	0	42	30
Colitis	50	7	0	30	13
Enteritis	35	22	4	3	6
Primary ventricular filling/overeating	27	21	2	3	1
Peritonitis	31	9	3	9	10
Small intestinal impaction	18	4	6	1	7
Verminous arteritis	23	3	2	8	10
Gastric ulceration	20	17	1	2	0
Descending colon : Enterolit/obstipation/volvulus/strangulation	10	3	2	2	3
Adynamic ileus	9	5	0	1	3
Muscularis hypertrophy	6	0	0	3	3
Grass sickness	6	2	0	2	2
Other	23	7	2	6	8
Totals	1588	946	141	147	354

**Table 3:** Survival in relation to location and type of lesion in colic horses treated at University Hospital for Large Animals (UHLA), Denmark 2000–2009

	**Survival ratio**	**Survival rate (%)**	**Standard Error (%)**	**95 % CI (%)**
*Location of lesion:*				
Large intestinal lesion	664/852	78	1.4	75; 81
Small intestinal lesion	79/230	34	3.1	28; 40
Type of lesion*:				
Strangulating	48/233	21	2.6	15; 26
Non-strangulating	1037/1281	81	1.1	79; 83

*Horses diagnosed with ruptured viscus are not included.

Among the cited studies in Table 1, the proportion of medical and surgical cases and the rates of euthanasia before treatment vary among the cited studies (range of 3% to 21%), p < 0.0001).

## Discussion

This study is the first to report the short term survival rates for all horses with colic admitted to a Danish university hospital. The overall short term survival rate of 68% was similar to those in some studies [3,5-7] but lower than those in others [8,9]. A number of reasons may account for these differences (see the outline in Figure 1), and some of these will be discussed in the following.

Because surgical colic cases generally carry a higher risk of mortality than medical colic cases, the proportion between medical and surgical cases in a hospital influences the overall outcome of colic. Among the cited studies in Table 1, the proportion of medical and surgical cases differed in a statistically significant way (p < 0.0001). Therefore the comparison of overall colic survival rates between the cited studies may not permit valid conclusions to be drawn. However, interestingly, there seem to be no obvious correlation between a high proportion of medical patients and a high short-term survival rate. A high proportion of surgical cases might indicate that the caseload was carefully selected by the referring veterinarian, and that only the most severe cases were presented at the hospital. On the other hand, a high proportion of surgical cases could also indicate that the hospital policy was biased toward surgery: to operate a proportion of colic horses that would have recovered without surgery. The decision to use surgery may in some cases be straightforward, based on history, clinical signs and paraclinical parameters. In other cases, the animal may need further diagnostic tests and observation – for example, horses with nephro-splenic entrapment, as a large proportion of these horses are known to recover without surgical intervention [10,11]. In contrast to this, if criteria for surgery are stricter in one hospital than another, the mortality rate might be higher as a result of the greater severity of the disease in the operated horses [12].

Also, diagnosis, localization and type of lesion play a role for the prognosis. From Table 3 it is seen that horses suffering from a small intestinal lesion had a lower short term survival (34%, CI: 28- 40%) than those with a large intestinal disease (78%, CI: 75-81%), regardless of whether the disease was medical or surgical. Likewise, strangulating lesions had a poorer survival rate than non-strangulating lesions. The survival rates reported here may be compared with Mezerova *et al*. [13] who found short-term survival rates after surgery involving the small intestine, the colon and the cecum to be 60%, 73% and 37%, respectively. Mair and Smith [14] identified corresponding rates of 64% in cases involving surgery of the small intestine, 57% for the caecum and 78% for the colon. Sutton *et al*. [8] obtained a 37% survival rate for surgical small intestinal lesions and a 74% survival rate in surgical cases with large intestinal lesions. The explanation for the relatively low short term survival for small intestinal diseases in the Danish cases is not clear, but may be related to the horse owners’ reluctance to choose surgery over euthanasia when a relatively poor prognosis is given in such cases. The choice of euthanasia based on the statistics of survival lowers the survival rate even more, as this choice eliminates the horses that would have survived. When a horse was allowed to recover from surgery, the chances of short-term survival were generally good, between 75% and 87% (Table 1). The variation in overall surgical survival rates may therefore be attributed to factors arising before and during surgery, rather than surgical skill or technique.

In the present study the single largest risk for mortality was euthanasia, since 59% of all horses recommended for surgery were euthanized, either before surgery or on the operating table, and 159 (10%) of the horses were euthanized without the recommended surgical treatment. From the owner information record and the medical record it appeared that this was not necessarily the horses with the worst prognoses. In some studies it was not possible to distinguish between euthanasia and other causes of death, and it was not always possible to determine when the decision to euthanize was taken, and in particular whether it was made before or after a diagnosis had been made. One would expect a relationship between a high euthanasia rate and high surgical short-term survival rate if only the most severe cases were euthanized. Interestingly, there was no such relationship, suggesting again that the choice of euthanasia was not related to severity.

The study showed that euthanasia is very complex factor affecting the use of survival as a success parameter [15]. Wide variations exist in attitudes to euthanasia. Sutton *et al.*[8] explicitly mention a cultural aversion to euthanasia among Muslim and Jewish populations as an explanation for low short-term survival rates. By contrast, in Denmark, discussions with owners suggest the decision to euthanize is affected by a pronounced concern that the horse should not suffer. Euthanasia is therefore often preferred as a way of ending suffering; this is sometimes so, even when suffering can be medically curtailed and believed to persist only for a limited period. No equine literature exist on this subject, and the present study therefore sought to retrospectively investigate the reasons for the observed high frequency of euthanasia by screening the information records where the communication between client and surgeon is recorded in writing, either by the surgeon or an assistant. During the screening procedure, it became obvious that the data obtained were of very heterogenic quality and therefore of limited use in this study. Most owners were in emotional distress during the decision making process, and in many instances it was impossible to discriminate between the effects of the owner’s financial constraints, the owner’s perception of the welfare of the horse, the owner’s plans to make future use of the horse, and the surgeon’s advice on the decision. Because many Danish horse insurance companies will not agree to compensate for the loss of a horse unless surgical treatment has been attempted, the insurance status could have played a major role in decisions both for surgery and euthanasia. We conclude that because of their effect on survival rates, attitudes towards suffering and euthanasia should be studied prospectively with standardized methods.

The present study shows that factors lying beyond the control of the clinician may influence the outcome of the hospital service. The continuous improvement of diagnosis, treatment and clinical skills requires a systematic approach, described as clinical governance [2,16]. The present study indicates that an agreed set of inclusion and exclusion criteria should be applied in retrospective studies if these studies are to be subjected to meta-analyses in the future. Also the analysis and development of risk stratification models – for example, using disease severity scores [17,18] – could be a productive next step. Finally, when mortality is the outcome measure being investigated, reasons for euthanasia must be investigated and defined.

In conclusion, this retrospective study of equine colic outcomes demonstrates that short-term survival rates were biased by the prevalence of euthanasia, and thus that it is difficult to use such rates as the sole criterion for evaluation of success. If survival rates are to be used as an indicator of the quality of care in colic treatment, and if they are to permit hospitals, techniques and surgeons to be compared, the use of disease severity scores and a predefined set of reasons for euthanasia will be needed in prospective studies.

## Competing interests

The authors declare that they have no competing interests.

## Authors’ contributions

MTC participated in collection and verification of data, carried out descriptive statistics and drafted the manuscript. ND participated in collection of data and carried out descriptive statistics. CK participated in collection of data and carried out descriptive statistics. KSBS participated in collection of data and carried out descriptive statistics. THP participated in the verification of the data and helped draft the manuscript. PHA conceived of the study, participated in its design and coordination, verification of data and helped draft the manuscript. All authors read and approved the final manuscript.
